# Public health spending in Sub-Saharan Africa: exploring transmission mechanisms using the latent growth curve mediation model

**DOI:** 10.1186/s13561-023-00472-5

**Published:** 2024-02-19

**Authors:** Wa Ntita Serge Kabongo, Josue Mbonigaba

**Affiliations:** School of Accounting, Economics and Finance, University of KwaZulu Natal, University Road, Westville Campus, Durban, South Africa

**Keywords:** Sub-Saharan Africa, Public health spending, Latent growth curve mediation, I18

## Abstract

In response to the imperatives of universal health coverage, structural factors that may hinder the effectiveness of increased spending in sub-Saharan Africa (SSA) need attention. This study assessed the mediating role of these factors in domestic general government health expenditure (DGGHE) effects to propose solutions for improving population health outcomes (PHO). The analysis used the Latent Growth Curve Mediation Model (LGCMM) approach within the structural equation model (SEM) framework for panel data from 42 SSA countries from 2015 to 2018. The findings were that malaria and female education formed a channel through which DGGHE imparted its effects on DALY in SSA, and these effects were achieved via the specific path from the DGGHE slope to the DALY slope, via malaria and female education slopes. However, the paper found no evidence of immunization coverage mediating the relationship between DGGHE and DALY in SSA. The paper concludes that structural factors affect the effectiveness of DGGHE on PHO, implying that governments should emphasize existing programs to fight against malaria and increase immunization coverage.

## Highlights

• Sub-Saharan Africa has many mediating factors acting as barriers to the effect of investments in health.

• The Latent Growth Curve Mediation Model is used to assist the understanding of such factors.

• The mediation effect (transmission mechanisms) of these factors must be understood for proper policymaking in favour of universal health coverage.

## Introduction

Government interventions across countries have wide implications for people's lives [86]. Stiglitz's view of government actions reflects the breadth of the debate over the effects of government decisions, which might take the form of interventions, such as public health spending (PHS) [78, 99]. Given the limitations of progressive taxation in achieving the desired level of income redistribution [56, 75], governments are increasingly achieving income redistribution through the expenditure side of the budget, particularly through social spending, including health spending [12].

Public health spending (PHS) is an example of an intervention targeted at increasing the quality and use of healthcare services to enhance population health outcomes (PHO). PHS is an important political tool for addressing concerns of equity in health care utilization, ensuring equitable income (re)distribution, reducing catastrophic health spending, and accelerating progress toward universal health coverage (UHC) [74, 104]. It also includes transfers from domestic government revenue, social insurance contributions, and compulsory prepayments [98].

The health funding needed to develop health is limited in the SSA region, which records the world's most acute health financing gap [87]. The African region accounted for only 1% of the global health spending in 2015, accounting for 23% of the global population and 23% of the disease burden [87]. Moreover, Africa's per capita expenditure on healthcare is one-tenth of the global average [68]. Moreover, the low health funding available in the region does not achieve the expected health outcomes [24, 54], as SSA countries are often related to the waste of health care resources, which impairs health systems' ability to deliver high-quality care and enhance health [27].

The region has experienced poor PHO for years and lags behind in achieving UHC and health-related sustainable development goals [90, 97]. PHO has barely improved in Africa in the last few decades and remains the worst compared to other regions. Healthy life expectancy (a measure of life expectancy adjusted for years spent with disability) has been rising in the region, rising from 50.9 to 53.8 years between 2012 and 2015, the largest increase in any WHO region, albeit from the lowest level. From 2000 to 2015, there was a significant reduction in disability-adjusted life-years (DALY) associated with the leading 10 causes of morbidity. This decline can be attributed to decreased prevalence of Malaria, HIV/AIDS, and diarrheal disorders. In 2019, the SSA region recorded approximately 215 million malaria cases and 386,000 malaria-related deaths. These figures accounted for about 90% of the world's cases and deaths [97]. In the SSA region, people between 30 and 70 years of age have a 20.7 percent chance of dying from one major noncommunicable disease (NCD). Moreover, the region has a high prevalence of all four key risk factors (tobacco use, drinking alcohol, physical inactivity and bad diets) for noncommunicable diseases, as indicated in the Global Action Plan for the Prevention and Control of Noncommunicable Diseases (2013–2020). The region's PHO weakness is a crucial issue because it harms the economy and wealth of countries while also exacerbating poverty.

Despite the potential role of PHS in improving PHO, there is currently a dearth of research on how PHS affects PHO in SSA owing to the complex interaction between PHS and PHO [22, 64]. Evidence in SSA indicates that interwoven and interrelated structural setting factors obstruct this relationship so that the effectiveness of health spending is generally affected [11, 41]. When dealing with such complex causal pathways, it is not enough to know whether interventions work, but there is a need to understand how they work [21]. Elucidating the process or pathway through which interventions affect outcomes is necessary because it responds to the above question and allows for devising appropriate interventions [29]. This insight can be gained through process analysis, which primarily sheds light on the mechanisms through which a programme affects an outcome.

Investigating the effectiveness of PHS on PHO in SSA, the majority of studies performed impact analyses, in which they assessed the ultimate effects of health spending on PHO [4, 7, 30, 31, 34, 65, 85, 95]. Nevertheless, limited studies have conducted the required process analysis to examine the complex causal relationship between PHS and PHO. One framework that addresses this relationship is the health field model, developed by Evans and Stoddart, which poses a dynamic analytical approach to understanding health [28]. The evidence indicates that PHO is not solely produced by a singular, isolated cause but rather by a series of causations in which an intricate interplay of prior events influences each connection [50]. Hence, to examine the change in PHS associated with a change in PHO within this complex framework, it is imperative to evaluate all factors along the PHS-PHO pathway, as they may influence this pathway. For example, when a government decides to increase PHS to improve PHO, this increase is made through funding activities that directly or indirectly impact PHO, such as financing medical care or health prevention activities. The latter's success, change, or improvement is due to other health system features, such as health literacy of patients and disease burden, contributing to PHO changes. Thus, for PHO to change, PHS must bring about changes in medical care or health prevention activities, but this could be affected by structural factors in bringing about changes in PHO. This study posits that variable changes can occur in either the initial level, growth rate, or both the initial level and growth rate. This differentiation of changes can provide significant insights into the relationship under investigation. Within the confines of this particular setting, the primary question arising from this investigation is whether the change over time in PHO is related to the change over time in PHS through changes over time in mediating factors.

Therefore, this study aims to assess whether structural factors serve as channels through which PHS impacts population health outcomes in SSA. The study evaluates the mediational processes in the relationship between DGGHE and PHO, focusing on the slope-slope mediating effect because it presents potential changes in specific countries [67]. This study applied the latent growth curve mediation model (LGCMM) within the structural equation model (SEM) framework for statistical analysis. This approach provides the benefit of distinguishing between variable changes in the initial level and growth rate, which may exhibit divergent trends. It also accounts for temporal precedence in the mediation process analysis while considering the possible endogeneity between health expenditure and PHO.

The remainder of this paper is organized as follows. The following section provides a brief review of the literature. Review of the literature section presents the theoretical framework and hypotheses development. Theoretical Framework and Hypotheses section presents the methodology used in this analysis. The study results are described and analyzed in Methodology section. Before the conclusion, a discussion of the results is conducted in Study results section.

### Review of the literature

Process analysis enables the identification and comprehension of problematic factors in causal relationships. In this process, the study employs the mediation analysis approach to clarify the mechanism through which interventions such as PHS affect outcomes such as PHO. Only a few studies in SSA have utilized mediation analysis to examine the link between PHS and PHO. For example, Makuta and O'Hare [52] used a sample of 43 SSA nations from 1995 to 2011 to analyze PHS's direct and indirect effects, using under-five mortality and life expectancy as PHO measures and fixed effects as the estimation technique. The study considered governance as the mediating factor in the association between PHS and PHO using the coefficient of the interaction term between PHS and governance as an indirect effect estimate. The findings suggest that the impact of PHS is mediated by the quality of governance [52]. These findings may suffer from soundness since the study used the interaction term to assess the mediation. In the literature on mediation and moderation, interaction terms are a statistical approach for assessing the moderating factor of intervention-outcome relationships [37, 91]. Using the interaction term to assess the mediating effect between variables was inappropriate.

A recent study by Mallaye and Yogo [53] considered a sample of 95 developing countries, including SSA countries, to investigate the direct and indirect effects of health aid on child mortality from 1990–to 2011. This study used a seemingly unrelated regression framework to test the mediating effects of health spending, female education, and governance on the relationship between health aid and child mortality. The standard mediation analysis based on a single mediator was conducted. The study findings suggest that the relationship between health aid and child mortality is fully mediated by female education, as measured by the female primary education completion rate and governance, as measured by both governance effectiveness and voice accountability [53]. This study conducted a single mediation, in which the temporal precedence of variables was ignored, as this could result in biased estimates [18]. Additionally, because the mediators were evaluated separately, there was a concern about biased estimates of indirect effects due to the exclusion of probable mediator interactions [92]. Another study by Okwan and Kovacs (2019) applied the partial least squares SEM techniques to cross-sectional data from 35 SSA countries over the period 2008–2015 to assess the causal relationships among maternal mortality, socioeconomic, socio-cultural and medical latent variables in SSA. The findings from the mediating effects investigation suggest that the latent medical variable significantly negatively mediates the relationship between the latent construct of socio-cultural determinants and maternal mortality [69]. The technique used to assess the mediation in this study can manage multiple mediations but is inappropriate for panel data and does not account for the temporal precedence of variables.

In contrast to previous studies, this study applied the LGCMM approach to investigate the mediational process between PHS and PHO in SSA countries. LGCMM is appropriate for panel data, supports the multiple mediation analysis, and considers the temporal precedence of factors. A parallel process latent growth curve modelling tool is widely used to assess change pathways in evaluating public health interventions [49, 76]. In this Model, the mediational process is modelled as the growths of intervention, mediator and outcome variables viewed as distinctive LGC [18, 60, 82]. To the best of our knowledge, no study in the SSA region has investigated the effectiveness of PHS in improving PHO using this approach.

### Theoretical Framework and Hypotheses

**Fig. 1:**
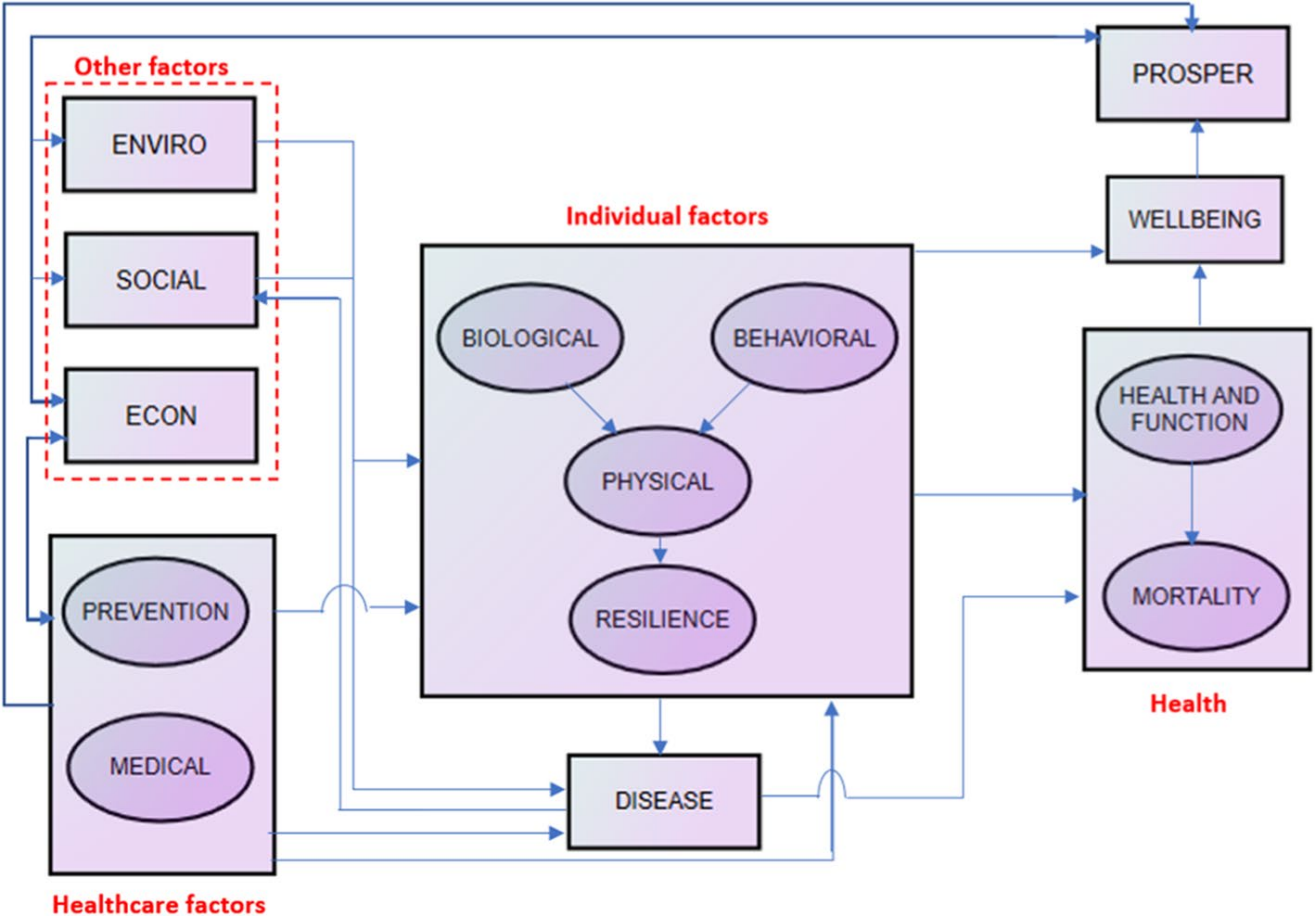
Study framework Note. This study framework was developed based on Health Field Model. Adapted from "Producing health, consuming health care", by Evans, R.G. and Stoddart, G.L., 2010, Soc Sci Med, 31(12), 1347–63, available: http://dx.doi.org/10.1016/0277-9536(90)90074-3

This study was based on the Health Field Model [28] to investigate the PHS pathways to PHO. The study used the modified version in Fig. 1, which shows the cyclic integration of health, economic wealth, and well-being. Globally, the Model suggests that improving health outcomes positively impacts well-being, which is necessary to promote prosperity and economic growth. Prosperity makes the government allocate parts of the achieved economic growth to social, economic, and environmental causes, while these causes also contribute to realising economic prosperity. For example, economic causes, including PHS, may improve health factors, individual elements, disease reduction, and health outcomes, and the cycle is achieved. An in-depth discussion of this Model is provided elsewhere [28].

This study provides all the potential paths through which the determinants of health affect health outcomes, including the hypothesized paths. Some segments on the paths indicated in the study framework have been investigated. This is the case for the segment between immunization and PHS [16, 23], segment immunization-PHO [51, 58, 89], segment PHS-malaria [57, 70, 79], segment malaria incidence-female education [14, 25, 73], and segment female education-PHO [10, 55, 84]

Following the study objective and framework and considering the segments mentioned above, this study developed the following hypotheses using the transmittal approach, which requires only a single hypothesis to assert that the relationship between the predictor and outcome is mediated by the mediator [77]. It is worth noting that the study measured PHS by DGGHE and PHO by DALY and under-five mortality, which, according to WHO, one unit represents the loss of an equivalent one year of full health. Malaria incidence and education as measured by female education) were used as mediators for mediational process 1, and immunization coverage was used as a mediator for mediational process 2.

- H1: The relationship between change in DGGHE growth rates and DALY growth rates is sequentially mediated by malaria and education growth rates (mediational process 1).
- H2: The relationship between DGGHE growth rates and DALY growth rates is mediated by immunization growth rates (mediational process 2).

### Methodology

#### Data

This study utilized secondary data obtained from 42 sub-Saharan nations spanning the period from 2015 to 2018, considering the availability of the data. The data above, sourced from the Global Health Data Exchange, World Development Indicators, World Governance Indicators, and Global Health Expenditures databases [40, 100–102], indicate a sample size of 42 observations throughout four distinct periods. Before analysis, all data were cross-checked for consistency with specific sources, such as the country's national health accounts reports. According to Duncan et al. [26], the sample size used in this study satisfies the requirement of a minimum of three time periods to investigate latent growth curves with confidence in the accuracy of the estimated parameters [26]. In addition, Hart and Clark [36] and Shi et al. [81] contended that, within the SEM framework, multivariate latent growth curve models, such as the LGCMM, could be applied with a very small (*N* = 42) non-normally distributed sample without the risk of type I errors [36, 81]. Of the data collected for this study, 3.5% were missing. A multiple imputation approach was used to address the issue of missing data.

The key study variables were DGGHE and DALY. The DGGHE represents the domestic resources that governments controlled overall and used as a crucial policy instrument for accelerating progress towards UHC and achieving health-related SDGs. The DALY is a health gap summary measure of population health that accounts for both mortality and morbidity to characterize the health of a specific population [32]. It has been most commonly used to assess the disease burden. It has proven useful to researchers and health professionals because it provides information on the population's disease burden and helps to identify priority health areas for implementing PHS interventions. The description and sources of the study variables are presented in Table 1.

**Table 1:**
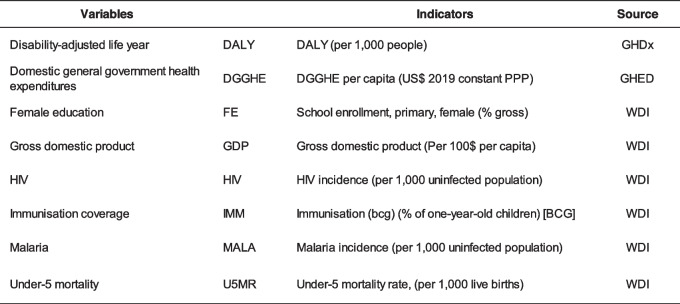
Variable description and source

*GHDx *Global Health Data Exchange, *GHED *Global Health Expenditures Database, *WDI* World Development Indicators.

#### Empirical Model and Estimation Technique

**Fig. 2:**
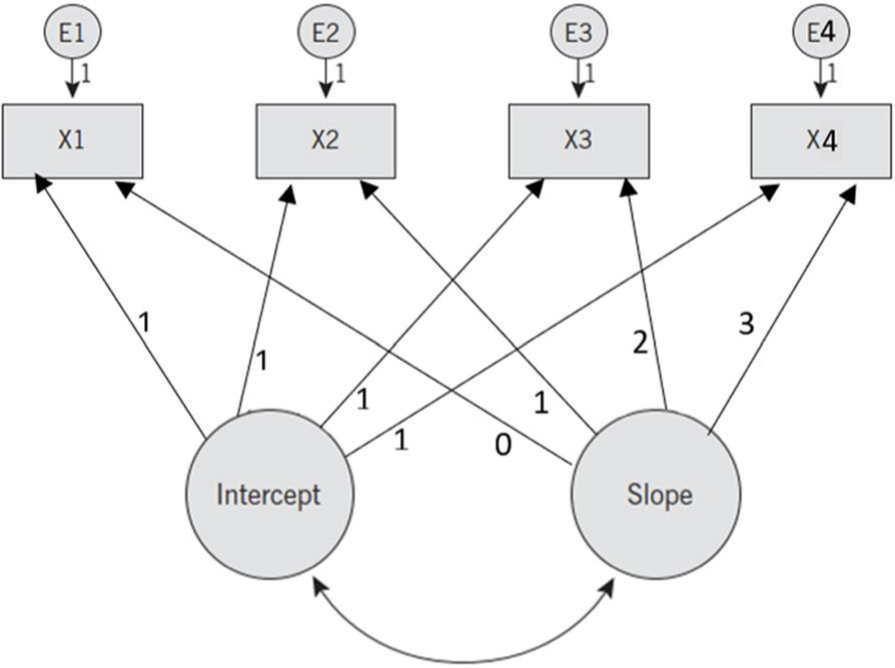
Latent linear growth model Note. X1 to X4 represent observed data collected over four periods, t = 1, 2, 3, and 4; E1 to E4 are time-specific errors. The simple arrows depict the link between the intercept and slope of the observed variables. Double arrows depict the link between growth factors (intercept and slope). Arrows indicate the intercept loading parameters

This study investigated the longitudinal mediation processes in the DGGHE-DALY relationship. Evidence suggests that longitudinal mediation models based on structural equation modelling (including cross-lagged panel, latent growth curve, and latent difference score models) depict mediation processes in longitudinal data more accurately [66, 67, 106]. This study selected the LGCMM because it allows researchers to investigate how increased DGGHE (intervention) affects the level (intercept) and change (slope) of DALY (outcome) over time by varying the level and change of the mediator [18]. The modelling of latent intercepts and slopes distinguishes this Model, which provides information on a population's mean change characteristics (intercepts and slopes) as well as individual differences in these characteristics (intercepts and slopes) increasing the accuracy of estimates. Furthermore, the factor loadings for each latent variable can be adjusted to account for different intercept and growth shape values. Each variable included in this study model reflected a univariate latent growth modelling (LGM) process, as illustrated in Fig. 2. which graphically depicts the latent linear growth model.

The observed X-scores depend on the latent intercept factor, latent slope factor (with factor loadings representing the predicted slope), and time-specific errors [46]. Because the intercept remains constant across time, the intercept factor loadings are always one. Because linear growth is expected, the slope factor loadings were 0, 1, 2, and 3. An observation at any time can be chosen as the intercept (i.e., the observation with a factor load of 0), and slope factor loadings can be modelled in various ways to reflect different patterns of change.

The general Equation related to the Model in Fig. 2 is expressed as follows:

1 $${y}_{ti}={\eta }_{0}+{\eta }_{1}{\lambda }_{t}+\left({\varsigma }_{0i}+{\lambda }_{t}{\varsigma }_{1i}+{\varepsilon }_{ti}\right)$$

where the observed repeated outcome measures ${y}_{ti}$ comprise the random (${\varsigma }_{0i}+{\lambda }_{t}{\varsigma }_{1i}+{\varepsilon }_{ti}$) and fixed (${\eta }_{0}+{\eta }_{1}{\lambda }_{t}$) components of the growth paths. The fixed-term represents the expected values of ${y}_{ti}$ at a specific time point *t*. The change in the growth trajectory is divided into within- and between-country changes and described as three unobserved sources of variations: ${\lambda }_{t}{\varsigma }_{1i}$ – between-country change in the rate of outcome change; ${\varsigma }_{0i}$ – between-country change in the initial level of outcome measure; and ${\varepsilon }_{ti}$ – within-country change in recurrent outcome measures. The random factors of the growth trajectory describe changes in country trajectories over time and across countries. The covariance between ${\varsigma }_{0i}$ and ${\varsigma }_{1i}$ illustrates the link between the initial outcome level and the rate of outcome change over time.

The study model consists of the following system of Eqs. (2, 3, 4, 5, 6, 7 and 8), representing the process of each study variable.

2 $${DALY}_{ti}={\eta }_{0}^{DALY}+{\eta }_{1}^{DALY}{\lambda }_{t}^{DALY}+\left({\varsigma }_{0i}^{DALY}+{\lambda }_{t}^{DALY}{\varsigma }_{1i}^{DALY}+{\varepsilon }_{ti}^{DALY}\right)$$

3 $${DGGHE}_{ti}={\eta }_{0}^{DGGHE}+{\eta }_{1}^{DGGHE}{\lambda }_{t}^{DGGHE}\left({\varsigma }_{0i}^{DGGHE}+{\lambda }_{t}^{DGGHE}{\varsigma }_{1i}^{DGGHE}+{\varepsilon }_{ti}^{DGGHE}\right)$$

4 $${MALA}_{ti}={\eta }_{0}^{MA}+{\eta }_{1}^{MA}{\lambda }_{t}^{MA}+\left({\varsigma }_{0i}^{MA}+{\lambda }_{t}^{MA}{\varsigma }_{1i}^{MA}+{\varepsilon }_{ti}^{MA}\right)$$

5 $${FE}_{ti}={\eta }_{0}^{FE}+{\eta }_{1}^{FE}{\lambda }_{t}^{FE}+\left({\varsigma }_{0i}^{FE}+{\lambda }_{t}^{FE}{\varsigma }_{1i}^{FE}+{\varepsilon }_{ti}^{FE}\right)$$

6 $${IMM}_{ti}={\eta }_{0}^{IMM}+{\eta }_{1}^{IC}{\lambda }_{t}^{IMM}+\left({\varsigma }_{0i}^{IMM}+{\lambda }_{t}^{IMM}{\varsigma }_{1i}^{IMM}+{\varepsilon }_{ti}^{IMM}\right)$$

7 $${GDP}_{ti}={\eta }_{0}^{GDP}+{\eta }_{1}^{GDP}{\lambda }_{t}^{GDP}+\left({\varsigma }_{0i}^{GDP}+{\lambda }_{t}^{GDP}{\varsigma }_{1i}^{GDP}+{\varepsilon }_{ti}^{GDP}\right)$$

8 $${HIV}_{ti}={\eta }_{0}^{HIV}+{\eta }_{1}^{HIV}{\lambda }_{t}^{DA}+\left({\varsigma }_{0i}^{HIV}+{\lambda }_{t}^{HIV}{\varsigma }_{1i}^{HIV}+{\varepsilon }_{ti}^{HIV}\right)$$

where DALY, DGGHE, MALA, FE, IMM, GDP, and HIV represent the disability-adjusted life year, domestic general government health expenditure, malaria incidence, female education, immunization coverage, gross domestic product, and HIV/AID, respectively. Each Equation from the system above relates to the study variable processes and contains the same terms as in Eq. (1).

The study constructed both unconditional and conditional models using evaluated latent variables. The unconditional Model assessed the associations among variables, whereas the conditional Model was employed to explore the mediational processes. The associative or unconditional Model builds on covariance to connect Eqs. (2, 3, 4, 5, 6, 7 and 8) and assess their relationship. In contrast, the conditional Model uses regression to establish the link between the variables (represented by Eqs. (2, 3, 4, 5, 6, 7 and 8) following the study hypotheses.

#### Data analysis

The hypotheses were empirically tested following the procedure outlined by Cheong et al. [18] and Cheong [17]. This approach suggested three steps of modelling and testing the mediational processes within the LGCM framework, which allowed for evaluating a parallel process in mediation analysis [17, 18]. Initially, the form of the growth trajectory for each process was examined. The primary objectives of this step were to ascertain whether the hypothesized trajectory form was compatible with the data and whether the growth rates of the variables varied. Upon examining the growth pattern of the time points for the data, it was anticipated that the growth of the study variables would differ.

The Model shown in Fig. 2 was used in this investigation. The factor loadings of the latent intercepts were set to one, as the initial factors were constant. The free factor loadings approach was used to compute the factor loadings of the latent slopes of all variables. This approach helped determine growth curves based on the data by capturing all possible individual growth curves, including growth curves with time-specific errors, and selecting the best fit for the data [13]. By allowing the loadings on the growth rate factor to vary, the researcher hypothesized that the effects of the initial intervention, such as increasing PHS through the implementation of health programmes, would not be as effective as the subsequent interventions.

In the second stage, the individual LGCMs assessed in the first step were combined into a single parallel process model based on the hypothesized relationships between growth factors. Before this phase, an unconditional LGCM was constructed to assess the degree of association between these latent constructs. Finally, the estimated magnitudes of the mediational effect and the standard error were computed. Standard error was used to determine the significance of the mediation effect and to construct confidence intervals for these latent constructs [17]. As suggested by Grace [33], this study addressed the endogeneity issue by correlating the DALY errors with the mediator errors while using the maximum likelihood mean–variance adjusted (MLMV) as the model estimator [33]. The comparative fit index (CFI), Tucker-Lewis index (TLI), and root mean square error of approximation (RMSEA) were used to evaluate the goodness of fit of each Model [38]. IBM SPSS software (version 26.0) was used for data preparation (IBM [39], and MPLUS software version 8.10) was used for analysis [61].

### Study results

#### Descriptive analysis

Table 2 presents descriptive statistics of the study variables. Each variable's average and standard deviation (SD) were calculated for each year. Over four years, 42 countries were observed, yielding 168 observations.

**Table 2:**
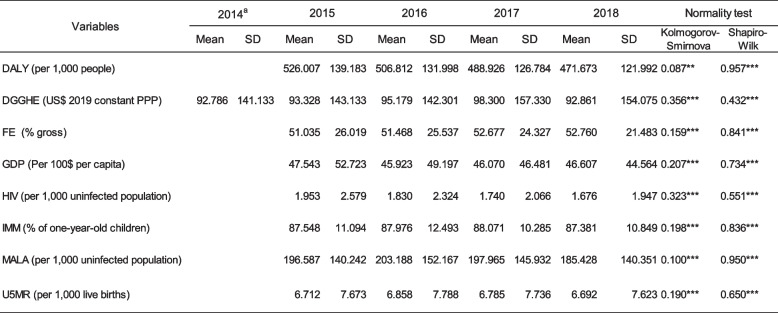
Descriptive statistics

^a^This year was considered to obtain the lagged DGGHE that helped to develop the main Model with lagged DGGHE. Source: Author's computed using the study dataset

**P < 0.05, ***P < 0.01

Throughout the study period, the mean value of the dependent variable (DALY) decreased from 526 to 476 per 1,000 people. Many decreases were attributable to the decline in mortality observed in the region, where countries such as Cabo Verde and Lesotho reported a decline in DALY from 264 to 260 per 1,000 people and from 927 to 857 per 1,000 people, respectively. The high SD observed in the region suggests that the DALY varied substantially between countries, owing to disparities in health. The predictor (per capita DGGHE) mean increased from $93.33 in 2015 to $95.18 in 2016 and $98.30 in 2017 before significantly decreasing to $93.86 in 2018. This fluctuation in DGGHE indicates a certain instability in public health resources, which makes forecasting the health sector challenging. Their high SDs ranged from 143.13 to 154.08, indicating that the DGGHE values varied significantly around their mean. This demonstrates the differences between nations.

Except for the FE variable, the means of the mediator (IMM, FE, and MA) and control (HIV and GDP) variables decreased throughout the study period.

#### Investigation of Growth Trajectories

The study fitted an unconditional linear LGC model for each variable, represented by a path diagram in Fig. 2. The results are displayed in Table 3, which includes the estimated growth trajectories, Model fit indicators, and intercept-slope covariances for each research variable. The intercepts were centred on the scores at the first time point for each Model, suggesting that the intercept represents the initial status of the growth curve. In addition, the estimated time scores for each variable's growth trajectory represent deviations from the linear time scores of [0, 1, 2, 3]. This suggests that all variables exhibited non-linear changes over time.

**Table 3:**
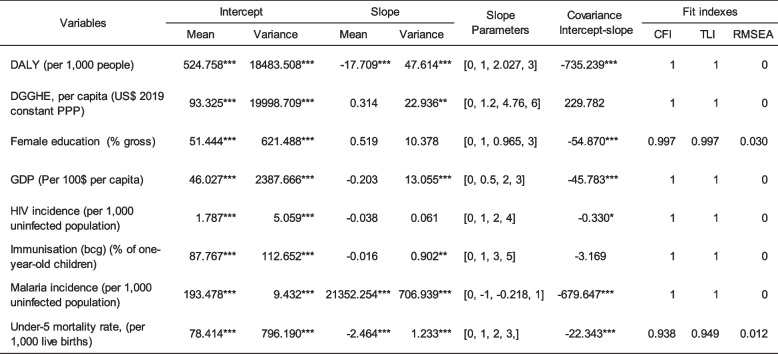
Estimates of growth trajectories

*CFI* Comparative fit index, *TLI *Tucker-Lewis index, and *RMSEA *Root mean square error of approximation. Source: Author 's computed using the study dataset and MPLUS 8.10 software. *p < 0.10; **p < 0.05; ***p < 0.01

The analysis of the results provides a comprehensive overview of the average DALY growth in SSA over time. The mean intercept term suggests that, on average, countries recorded a rate of 525 DALYs per 1,000 individuals in 2015. There exists notable variation at the country level around the mean intercept (*p* < 0.01). The mean latent slope suggests that DALY in SSA had a rapid annual drop of 17.7% throughout the study period. Furthermore, significant variability is observed at the country level around the mean slope (*p* < 0.01). The RMSEA estimate is lower than the threshold of 0.08 [48], the TLI estimate approaches the norm of 1, and the CLI estimate is higher than the threshold of 0.95 [35]. These results suggest that all the univariate DALY models fit the data well LGC fit the data. The alternative dependent variable, U5MR, exhibited almost the same characteristics as those of DALY. That is a large intercept variance, negative mean slope, and negative intercept-slope covariance. However, it differs from the DALY in its low slope variance.

The mean and variance of the DGGHE intercept were significant at all levels. The average intercept of DGGHE was 93.325 $ per capita, suggesting that, on average, sub-Saharan governments spent 93.33 USD per person. However, the high intercept variance of 19,998.71 shows a significant disparity between SSA countries. As an illustration, certain countries, such as the Democratic Republic of Congo (DRC), with a per capita DGGHE of $5.53 and Guinea-Bissau, with a per capita DGGHE of $11.61, exhibit a much lower value compared to other countries, such as South Africa (RSA) with a per capita DGGHE of $553.94 or Botswana with a per capita DGGHE of $700.88, which exceeds the former by more than 100 times. The variance of the DGGHE slope was statistically significant at the 5% level. This finding suggests that there is significant variation in DGGHE levels within countries in the sub-Saharan region. During the study period, countries such as Angola, Chad, the Republic of Congo, Guinea, Liberia, Madagascar, Sao Tome, Sierra Leone, and Zimbabwe exhibited DGGHE variations ranging from simple to nearly double. The main factors contributing to the variation in DGGHE in SSA countries include the prevalence of chronic diseases [96], urbanization that comes with factors that exacerbate health issues [1, 105], and public debt, which leads to increased government debt service costs, thereby reducing the resources available for general expenditures, including DDGHE [9]. With CFI, TLI, and RMSEA values of 1.000, 1.000, and 0.00, respectively, the estimated univariate DGGHE model fit the data well.

The intercept means, and variances of the control and mediating variables are significant at all levels. All control and mediator variables' means of the slopes were statistically insignificant, except for malaria. Only the variances of the slopes of GDP, HIV/AIDS, and immunization were statistically significant at all levels, the 5% level and the 10% level, respectively. The Model fit indices in Table 3 show that the univariate models of all control variables and mediators fit the data well.

#### Model estimates

Table 4 presents the descriptive Model fit indices for the unconditional Model that links the LGCMs analyzed in the previous section.

**Table 4:** The goodness of fit indicators of conditional and unconditional LGCM

Goodness of fit Indicator	Unconditional LGCM
RMSEA	0.050
CFI	0.968
TLI	0.961

CFI, TLI, and RMSEA stand for the comparative fit index, Tucker-Lewis index, and root mean square error of approximation, respectively. Source: Author 's computed using the study dataset and MPLUS 8.10 software

The Model fit indices in Table 4 indicate that the Model is a good fit for the data [15, 44]. Following this, conditional models were developed to analyze the mediational processes.

This study developed three models combining Mediational Processes 1 and 2 to achieve the study objective. The combination of mediational processes has been advocated in the literature because it accounts for the potential interactions of mediators [92]. The 1st, Main Model, was developed to evaluate the hypotheses. The 2nd was developed to consider the eventuality of lagged DDGHE. The 3rd used U5MR as an alternative dependent variable to the DALY, which the Global Burden of Disease Database modelled. The introduction of another health variables U5MR aimed at assessing the sensitivity of the results to the health indicator used. Table 5 presents the indirect, direct, and total effects estimates for the three models and their goodness-of-fit indicators.

**Table 5:**
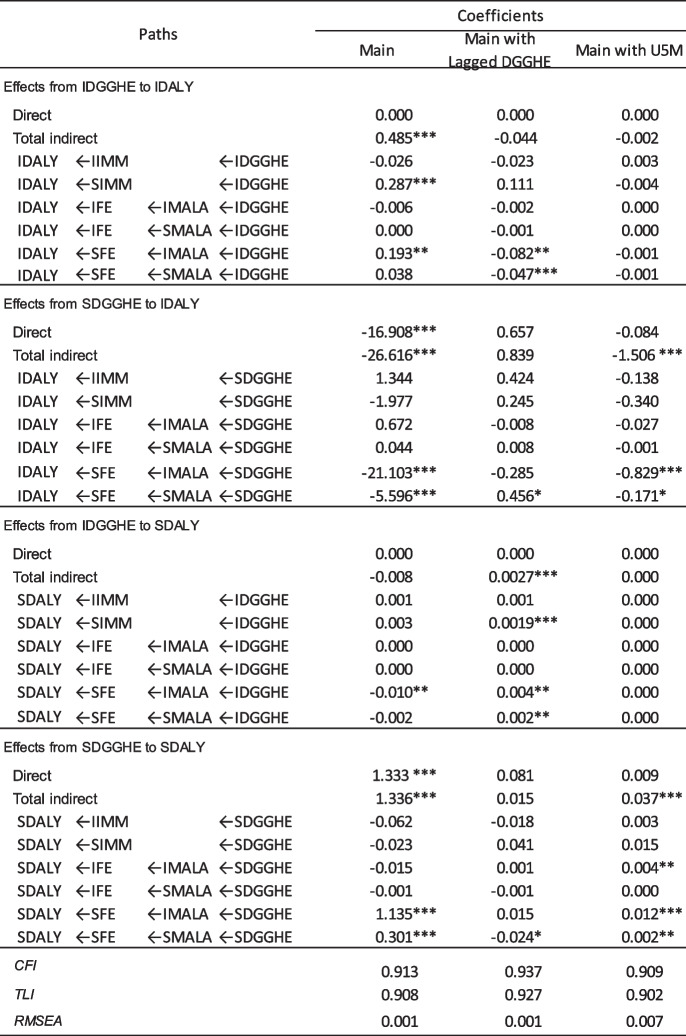
Indirect, direct and total effects estimates

*CFI *Comparative fit index, *TLI *Tucker-Lewis index, and *RMSEA *Root mean square error of approximation, IDALY = intercept DALY; SDALY = slope DALY; IDGGHE = intercept DGGHE; SDGGHE = slope DGGHE; IFE = intercept FE; SFE = slope FE; IHIV = intercept HIV; SHIV = slope HIV; IMALA = intercept MALA; SMALA = slope MALA; IGDP = intercept GDP; SGDP = slope GDP; IIMM = intercept IMM; SIMM = slope IMM. Source: Author 's computed using the study dataset and MPLUS 8.10 software. *p < 0.10; **p < 0.05; ***p < .01

Table 5 shows the four main pathways involving the two mediation processes by which DGGHE factors affect DALY factors. These main pathways are from IDGGHE to IDALY and SDALY and from SDGGHE to IDALY and SDALY. They included 24 pathways investigated, eight of which were related to mediational processes 2 and 16 to mediational processes 1. The findings presented in Table 5 show that certain main pathways, specifically those originating from SDGGHE to SDALY (in the Model incorporating delayed DGGHE) and from IDGGHE to SDALY (in both the main Model and the Model incorporating U5MR), exhibit an overall indirect effect that is not statistically significant. However, it is worth noting that certain specific pathways within these models do exhibit a statistically significant indirect effect. The findings presented in this table were used in the subsequent section to assess the mediation processes being examined. The Model fit indices presented in Table 5 suggest that the three conditional LGCMs fit the data well [38].

#### Mediation assessment

Two mediational processes were simultaneously investigated in the relationship between DALY and DGGHE. Based on the study hypotheses, the intercept-intercept, intercept-slope, and slope-slope mediating effects were analyzed [93], focusing on the slope-slope mediating effects which present potential changes in individual countries [67].

Table 6 provides the estimates of the pathway coefficients of the Main Model. The results in Table 6 show statistically significant indirect effects in all pathways except for the pathways from IDGGHE to SDALY. The indirect effects suggest partial mediation in two pathways (SDGGHE to IDALY and SDGGHE to SDALY). The pathway from IDGGHE to IDGGHE suggested full mediation.

**Table 6:**
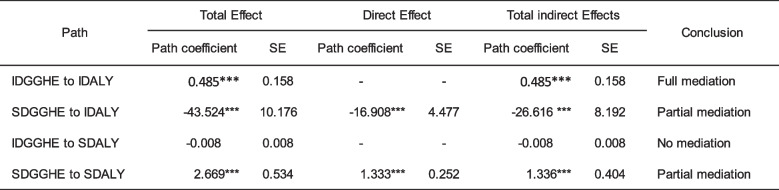
Estimates of pathway coefficients of Main Model

*CFI *Comparative fit index, *TLI *Tucker-Lewis index; and *RMSEA *Root mean square error of approximation; IDALY = intercept DALY; SDALY = slope DALY; IDGGHE = intercept DGGHE; SDGGHE = slope DGGHE. Source: Author 's computed using the study dataset and MPLUS 8.10 software. ***p < 0.01

**Fig. 3:**
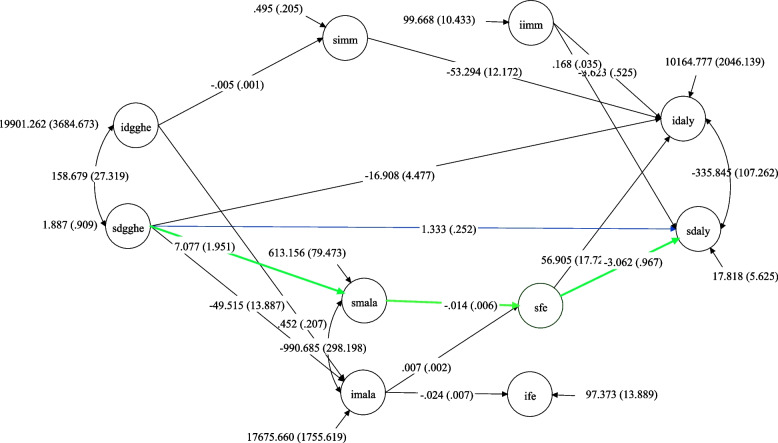
Main Model Mediation Pathways Note. Only the pathways with statistically significant paths are displayed. Covariates (GDP and HIV/AIDS) and factor loadings are omitted for simplicity. Green lines present mediation pathway and the blue line presents the direct effect path. Source: Author's computed using the study dataset and MPLUS 8.10 software

Focusing on the slope-slope mediating effects, the results indicate that the global trajectory from SDGGHE to SDALY presents a significant direct effect (1.333; *p* < 0.000) and a significant total indirect effect (-1.336; *p* < 0.001). The results in Table 6 suggest that this indirect effect results from the specific trajectories SDGGHE-SMALA-SFE-SDALY (-0.301; *p* < 0.001) and SDGGHE-IMALA-SFE-SDALY (1.135; *p* < 0.001). Highlighting the slope-slope mediating effects, the results in Table 6 demonstrate a partial mediation process, as evidenced by the statistical significance of the direct effect. The findings suggest that in the context of SSA, the association between the growth rates of DALY and the growth rates of DGGHE is sequentially and negatively mediated by the growth rates of malaria incidence and education. The results are visually presented in Fig. 3, which presents the Main Model mediation pathways, whereby the pathway of interest is depicted by green arrows, indicating the indirect effect, and a blue arrow, representing the direct effect.

This study attempted to account for the time delay in the Model by including the DGGHE with a lag of one period under the assumption that the impact of the DGGHE on DALY occurs after a one-year interval. Table 7 displays the outcomes of pathway estimation of the Model with lagged DGGHE.

**Table 7:**
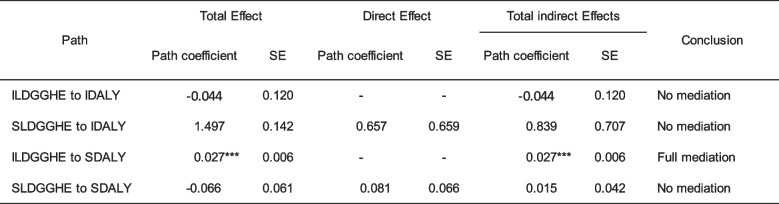
Estimates of pathway coefficients of Model with lagged DGGHE

*CFI *Comparative fit index, *TLI *Tucker-Lewis index; and *RMSEA *Root mean square error of approximation. IDALY = intercept DALY; SDALY = slope DALY; ILDGGHE = intercept lagged DGGHE; SDGGHE = slope lagged DGGHE. Source: Author 's computed using the study dataset and MPLUS 8.10 software. ***p < 0.01

Focusing on slope-slope mediating effects, the results indicate that for the global trajectory from SDGGHE to SDALY, the direct and indirect effects are not statistically significant. However, the detailed results in Table 6 indicate that the specific trajectories were SDGGHE-SMALA-SFE-SDALY (-0.024; *p* < 0.085). Highlighting the slope-slope mediating effects, the results in Table 6 show a full mediation process, as evidenced by the statistical non-significance of the direct effect. The findings suggest that in the context of SSA, the association between the growth rates of DALY and the growth rates of DGGHE is sequentially and negatively mediated by the growth rates of malaria incidence and education.

The outcome variable used in this study, Disability-Adjusted Life Years, was derived from a model developed by the Global Burden of Disease Database [59]. Examining the influence of a policy change on a modelled measure may provide challenges because of the potential for the policy change to already be inherently incorporated in the model results [6]. To address this potential concern, the present study examined an additional Public Health Service (PHS) indicator, namely, the Under-5 Mortality rate (U5MR), to corroborate the findings observed in the main Model. Table 7 displays the outcomes of the pathway estimation of Model with U5MR.

The results in Table 8 indicate a statistically significant indirect effect on the global trajectory from SDGGHE to SDALY. The results in Table 6 suggest that this indirect effect resulted from the specific trajectories SDGGHE-SMALA-SFE-SDALY (-0.002; *p* < 0.008), SDGGHE-IMALA-SFE-SDALY (0.012; *p* < 0.007), and SDGGHE-IMALA-IFE-SDALY (0.004; *p* < 0.012). Highlighting the slope-slope mediating effects, the results in Table 6 demonstrate a full mediation process, as evidenced by the statistical non-significance of the direct effect. The findings suggest that in the context of SSA, the association between the growth rates of DALY and the growth rates of DGGHE is sequentially and negatively mediated by the growth rates of malaria incidence and education.

**Table 8:**
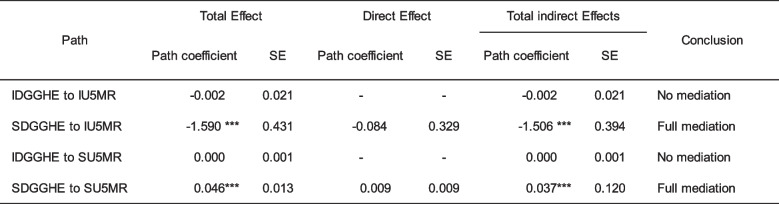
Estimates of pathway coefficients of Model with Under-5 Mortality rate

*CFI *Comparative fit index, *TLI *Tucker-Lewis index; and *RMSEA *Root mean square error of approximation. IU5MR = intercept U5MR; SU5MR = slope U5MR; IDGGHE = intercept DGGHE; SDGGHE = slope DGGHE. Source: Author 's computed using the study dataset and MPLUS 8.10 software. ***p < 0.01

The sensitivity analysis procedures available in the literature for multiple mediations hypothesize the independence between mediators [42, 43, 88]. This study incorporated numerous mediators in response to anticipation of possible interactions. Consequently, to evaluate the sensitivity of the findings, this study conducted an assessment of the reliability by investigating the impact of changes in the sample size [80]. To restrict the range of DALY values, the sample was subjected to exclusion of four observations with the most extreme DALY values. As a result, there was a reduction in the number of nations considered in the analysis, declining from 42 to 38. The recently estimated Model exhibited a robust level of fit (CFI = 0.944, TLI = 0.930, RMSEA = 0.07), meeting the acceptable standards set out by Jöreskog and Sörbom (1993). The updated Model revealed significant indirect effects for the specific SDGGHE-SMALA-SFE-SDALY pathway, with effect sizes of -3.749 (*p* < 0.01). The findings obtained were similar to those generated from the main Model and did not alter the information about mediational processes. This finding implies that the indirect effects of the study are robust as they remain unaltered by fluctuations in the number of observations.

## Discussion

This study examined how PHS affects population health outcomes in sub-Saharan countries. To this end, this study examined the transmission mechanisms of PHS in the relationship between PHS and population health outcomes, focusing on changes in variables over time. The study used DGGHE and DALY growth factors to measure PHS and population health outcomes, respectively and evaluated the simple and sequential mediations in the relationship between DGGHE and DALY growth factors by applying a parallel process within an LGCMM framework. We used data from a panel of 42 sub-Saharan countries from 2015 to 2018.

The research results suggested that malaria and female education formed a channel through which DGGHE imparts its effects on the DALY in sub-Saharan countries, and these effects were achieved via specific paths from the DGGHE slope to the DALY slope, via malaria and female education slopes. However, the study found no mediating effects of immunization on the relationship between DGGHE and DALY in sub-Saharan countries.

This study also identified mediating effects between DGGHE and DALY growth factors. For example, these were identified in the pathways from the DGGHE intercept to the DALY intercept via the malaria incidence and female education slopes, the DGGHE intercept to the DALY slope via the malaria incidence and female slope education slopes, and the DGGHE slope to the DALY intercept via the malaria incidence and female education slopes. These pathways involving the initial values of the growth factors were not the focus of our study, which looked at how the variables changed over time.

While the results of the study revealed a negative indirect effect of 0.451 of DGGHE caused by malaria and female education in the relationship between DDGHE and DALY, they also revealed that there was no direct effect, implying that the mediation of malaria and female education in the relationship between DGGHE and DALY was complete, that is, a full mediation [63].

These results also suggest that during the study period in SSA, the DGGHE slopes were inversely related to the DALY slopes via sequential changes in malaria and female education rates of change. This conclusion is consistent with Hypothesis 1, which states that malaria and female education growth rates serially mediate the relationship between DGGHE and DALY growth rates in sub-Saharan countries. Although the results support Hypothesis 1, they do not support Hypothesis 2, which states that immunization growth rates mediate the relationship between DGGHE and DALY growth rates in sub-Saharan countries.

The results indicated that malaria and female education were conjointly transmission channels of DGGHE, suggesting that increased DGGHE growth rates were negatively related to malaria growth rates, and malaria growth rates adversely affected female education growth rates, which in turn positively affected DALY growth rates. As a result, a total indirect effect of DGGHE growth rates on DALY growth rates was observed.

These conclusions were expected in SSA, where the constant fight against malaria is made possible by increasing DGGHE, and persistently decreasing malaria incidence. Thus, the continuing decline in malaria positively affected the school enrolment rate for girls. In turn, the increase in school enrolment enables the population to acquire the knowledge needed to understand and manage health issues, enabling them to remain healthy at all times, thus avoiding disease. The country-level impact improved health outcomes, such as reduced DALY [83]. For example, in countries such as Mozambique and Kenya, the reduction in malaria cases has led to an improvement in school results, which, in turn, has improved (decreased) the burden of disease [19]. Moreover, in SSA, the increase in DGGHE necessitated by higher immunization rates has indirectly ensured that disease prevention maintains a low DALY [8].

The pathways addressed in this study have not been explored in previous studies. However, despite the lack of direct research on the entire pathway, the conclusion derived from analyzing each segment of these pathways is theoretically supported by evidence from the existing literature. For example, Omoruyi [70] and Sede and Nosakhare [79] investigated the segment from the DGGHE slope to the malaria incidence. Their results suggested that increased PHS, which was measured by DGGHE in the current study, substantially contributed to the reduction in malaria cases in the African region, including SSA [70, 79].

Several studies have provided evidence of a relationship between malaria incidence and education, including education for females. The results of these studies revealed that malaria has significant negative effects on education, including female education, with the adverse effects of malaria ranging from school absenteeism [3, 10, 47] to attention deficits and cognitive dysfunction in children [20, 62, 71]; thus, the need to fight malaria.

The relationship between education, including female education, and population health outcomes has also been investigated, and the results indicated that female education has a beneficial influence on the health of the population, as measured by maternal health, neonatal mortality, under-five, and infant mortality [5, 45, 94, 103]. However, to the best of our knowledge, the current study is the first conducted in SSA to examine the transmission mechanisms of PHS by using growth factor variables and LGCMM.

A policy recommendation is for government initiatives in the medical sector to influence transient variables that greatly impact health outcomes. To reduce mortality and morbidity, for instance, governments could initiate interventions affecting factors, such as the burden of malaria and female education, that can potentially reduce mortality and morbidity conjointly, as measured through the DALY [2].

The primary contribution of the current study to the PHS literature on SSA is the investigation of mediational processes in the relationship between DGGHE and DALY. This could reveal the channel through which DGGHE affects the DALY. Other contributions include using the LGCMM technique and implementing a multiple mediation approach.

This study aimed to examine the transmission mechanisms to assess the effectiveness of PHS. This method employs an analysis of the mediation process considering the temporal precedence. Additionally, it considered the possibility that any change in the variable could be decomposed into a change in the initial level and a change in the growth rate, with these two components potentially having opposite directions [72]. This method has the potential to identify sustainable changes in variables that are consistently associated with changes over time and to determine whether initial levels can predict growth rates.

Another contribution of the study was the implementation of multiple mediation analyses that accounted for potential interactions between mediators and avoided biased estimates of indirect effects resulting from a separate evaluation of mediating effects [92].

However, the study had several limitations, including using a short time frame that only allowed data to be collected over four years. Consequently, the underlying transformation process may have been inadequately stated. Future studies should incorporate more time points to increase data quality and outcomes.

The free factor loadings approach was used for the study constructs to estimate the univariate LGCM. To allow the replication of the study, future investigations will need to use a set of factor loadings expressing a precise functional form [72].

Finally, DALY, used as a health indicator, only considers the negative elements of population health. Health indicators such as HALE, which capture the positive aspects of health, may be used. Nevertheless, this and the other abovementioned limitations should not undermine the research results. Instead, they should be viewed as groundwork for future research.

## Conclusions and policy recommendations

Despite the long-standing debate on the effectiveness of PHS in the SSA region, there is little evidence of the effects of PHS on PHO. This study adds to the existing literature by exploring the pathways through which PHS measured by DGGHE affects PHO measured by DALY to provide insights into the controversial link between PHS and PHO. Accordingly, this study applied the LGCMM with the MLMV estimator to panel data from 42 SSA countries for 2015–2018. The findings suggest that, on average, in SSA countries, higher growth rates of DGGHE are related to lower growth rates of DALY through the mediating effects of growth rates of malaria incidence and education. A key policy implication drawn from the study findings is that governments in SSA countries, in focusing on their redistributive role through DGGHE to improve PHO, should consider directing their interventions toward fighting malaria incidence. Therefore, in policies aimed at improving PHO in SSA, governments and policymakers should consider emphasizing existing programs for the fight against malaria. This would contribute to improving health outcomes through funding.

## Data Availability

The data that support the findings of this study are available upon request from one of the authors, WSK Email: sergekwn@yahoo.fr; + 15,196,946,179.

## Abbreviations

CFI: Comparative fit index
DALY: Disability-adjusted life year
DGGHE: Domestic general government health expenditures
FE: Female secondary education
GDP: Gross domestic product
HIV: HIV/AIDS incidence
IDALY: Intercept DALY
IDGGHE: Intercept DGGHE
IFE: Intercept FE
IGDP: Intercept GDP
IHIV: Intercept HIV
IIMM: Intercept IMM
IMALA: Intercept MALA
IMM: Immunization
LGCM: Latent Growth Curve Model
LGCMM: Latent Growth Curve Mediation Model
MALA: Malaria incidence
ML: Maximum likelihood
MLMV: Maximum likelihood mean–variance adjusted
PHO: Population health outcomes
PHS: Public health spending
RMSEA: Root mean square error of approximation
RMSEA: Root mean square error of approximation
SDALY: Slope DALY
SDGGHE: Slope DGGHE
SEM: Structural Equation model
SFE: Slope FE
SGDP: Slope GDP
SHIV: Slope HIV
SIMM: Slope IMM
SMALA: Slope MALA
SSA: Sub-Saharan Africa
TLI: Tucker-Lewis index
UHC: Universal health coverage
UNAIDS: The Joint United Nations Programme on HIV/AIDS
WDI: World development index
WHO: World Health Organisation
